# ﻿Three new species of Batrisini (Coleoptera, Staphylinidae, Pselaphinae) from southeast Xizang, China

**DOI:** 10.3897/zookeys.1228.143710

**Published:** 2025-02-21

**Authors:** Yong-Qin Zhang, Zi-Wei Yin

**Affiliations:** 1 Laboratory of Systematic Entomology, College of Life Sciences, Shanghai Normal University, Xuhui District, Shanghai 200234, China Shanghai Normal University Shanghai China

**Keywords:** Ant-loving beetles, *Arthromelodes*, distribution, new taxa, taxonomy, *Tribasodites*

## Abstract

Three new species of the ant-loving beetle tribe Batrisini, Reitter, 1882 (Pselaphinae: Batrisitae) from southeast Xizang, China are described: *Arthromelodeslhunzensis***sp. nov.**, *Tribasoditesliangi***sp. nov.**, and *Tribasoditesyumaicus***sp. nov.** Illustrations of the habitus and diagnostic features of these species are provided to aid identification. With these additions, the number of batrisine species known from Xizang increases to 83.

## ﻿Introduction

A recent monograph on the pselaphine tribe Batrisini Reitter, 1882 of Xizang has unveiled a previously undocumented yet diverse fauna, describing two new genera and 68 new species primarily distributed along the Himalaya and its southeastern region (Yin 2022). In this study, we report three new species belonging to the genera *Arthromelodes* Jeannel, 1954 and *Tribasodites* Jeannel, 1960, collected by Prof. Hong-Bin Liang and his team from a previously unsampled valley in Yümai, Xizang, China. These new findings increase the number of known Batrisini species in the region to 83 and highlight its potential for future discoveries, particularly in remote, underexplored areas.

## ﻿Material and methods

The material treated in this paper is deposited in the Insect Collection of Shanghai Normal University, Shanghai, China (SNUC), and the Institute of Zoology, Chinese Academy of Sciences, Beijing, China (IZCAS). The label data of the material are quoted verbatim. Dissected parts were mounted in Euparal on plastic slides pinned with the specimen. The habitus images of the beetles were taken using a Canon EOS R5 camera, equipped with a 7.5× Mitutoyo M Plan Apo lens, with a Raynox DCR-150 macro lens in between serving as the tube lens; and three 10W LED bulbs (5500 K) were used as the light source. Images of morphological details were produced using a Canon G9 camera mounted to an Olympus CX31 compound microscope under reflected or transmitted light. Helicon Focus Pro v. 8.2.0 was used for image stacking. All images were modi­fied and grouped into plates using Adobe Photoshop CC 2020.

Measurements were taken as follows: total body length was measured from the anterior margin of the clypeus to the apex of the abdomen; head length was measured from the anterior margin of the clypeus to the head base, excluding the cervical constriction; head width was measured across the eyes; the length of the pronotum was measured along the midline, the width of the pronotum equals the maximum width; the length of the elytra was measured along the suture; the width of the elytra was measured as the maximum width across both elytra; the length of the abdomen is the length of the dorsally exposed part of the abdomen along its midline, the width is the maximum width. The terminology follows Chandler (2001) and Yin (2022). Abdominal tergites and sternites are numbered in Arabic (starting from the first visible segment) and Roman (reflecting true morphological position) numerals, e.g., tergite 1 (IV), or sternite 1 (III). Paired appendages in the descriptions are treated as singular.

## ﻿Taxonomy

### ﻿ Arthromelodes lhunzensis sp. nov.

Taxon classificationAnimaliaColeopteraStaphylinidae

98BA89D2-B647-53EC-AF84-3D1053607EC2

https://zoobank.org/BBDF3698-E89C-4C79-A7B8-C80E7E0280A2

**Figure 1.**
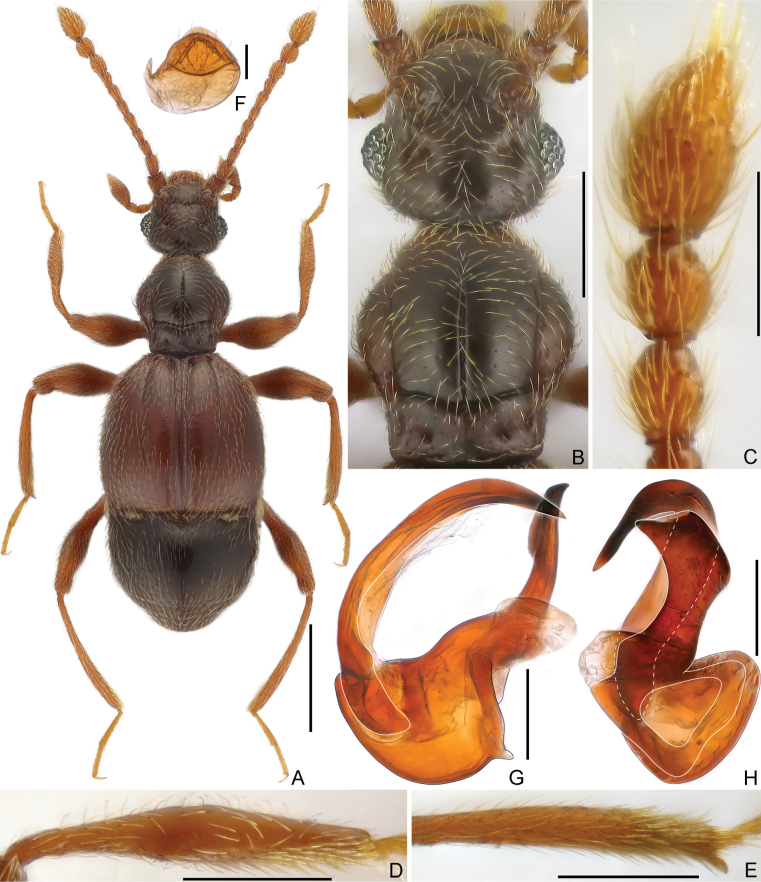
Morphology of *Arthromelodeslhunzensis* sp. nov., male **A** dorsal habitus **B** head and pronotum **C** antennal club **D** protibia **E** mesotibia **F** sternite 7 (IX) **G, H** aedeagus, lateral (**G**), and ventral (**H**). Scale bars: 0.5 mm (**A**); 0.3 mm (**B**); 0.2 mm in (**C, D, E**); 0.1 mm in (**F, G**); 0.05 mm (**A**).

Figs 1 , 4 Chinese common name: 隆子丽蚁甲

#### Type material

(1 ex.). ***Holotype***: China: • ♂: ‘China: Xizang, Shannan, Lhünzē County, Yümai Town, Jianzhejinzhe, 28°30'21"N, 93°07'07"E, 2930 m, in wood, 5.ix.2023, Hong-Bin Liang leg., 西藏隆子县玉麦乡件哲金哲’ (SNUC).

#### Diagnosis.

**Male.** Body elongate, length 2.1 mm. Head, pronotum and abdomen much darker in color than elytra. Head sub-rectangular; vertex finely punctate, with transverse sinuate sulcus between antennal tubercles, foveae asetose. Antenna elongate, antennomeres more or less elongate, lacking obvious modifications; antennomere 11 approximately as long as 9 and 10 combined. Discal striae of elytra extending to approximately apical 4/5 of elytral length. Protibia with shallow disc-like impression at apical 2/5 of ventral surface; mesotibia with distinct apical spine. Abdomen with large tergite 1 (IV) longer than tergites 2–4 (V–VII) combined in dorsal view, simple. Aedeagus strongly asymmetric, median lobe with moderately large basal capsule and subtriangular foramen, ventral stalk much shorter than dorsal lobe. **Female.** Unknown.

#### Description.

**Male.** Body (Fig. 1A) length 2.10 mm; head, pronotum and abdomen darkish-brown, elytra and legs reddish-brown, tarsi and mouthparts lighter in color. Dorsal surface finely punctate, covered with short pubescence.

Head (Fig. 1B) sub-rectangular, rounded at base, slightly wider than long, length 0.39 mm, width across eyes 0.41 mm; vertex finely punctate, with widely separated foveae (dorsal tentorial pits), with short, transverse sinuate sulcus at apical portion, lacking mediobasal carina; tempora slightly shorter than eyes, convergent posteriorly; antennal tubercles moderately raised; frons slightly impressed medially, confluent with clypeus; clypeus smooth, anterior margin carinate and moderately raised; ocular-mandibular carinae complete, distinct. Venter with small gular foveae (posterior tentorial pits) in single pit, with distinct median carina extending from pit anteriorly to mouthparts. Compound eyes prominent, composed of approximately 32 ommatidia. Maxillary palpus with palpomere 1 minute, 2 elongate, curved, pedunculate basally and enlarged apically, 3 short, sub-trapezoidal, 4 fusiform, widest near middle. Antenna moderately elongate, length 0.99 mm; club loosely formed by enlarged apical three antennomeres (Fig. 1C); antennomere 1 thick, subcylindrical, 2–7 each elongate, successively longer, 8 shortest, 9 much longer and broader than 8, 10 broader than 9, 11 longest, approximately as long as 9 and 10 combined (1:1), subfusiform.

Pronotum (Fig. 1B) slightly longer than wide, length 0.48 mm, width 0.44 mm, widest at middle; lateral margins rounded; disc moderately convex, finely punctate, with median longitudinal sulcus slightly longer than semicircular lateral sulci in dorsal view; lacking median antebasal fovea, with complete, deep transverse antebasal sulcus connecting lateral antebasal foveae; outer and inner pair of basolateral foveae distinct. Prosternum with basisternal (precoxal) portion longer than procoxal rests; with small lateral procoxal foveae; hypomeral grooves moderately long, extending from base anteriorly for almost entire length of hypomeron, lacking lateral procoxal pits, hypomeral ridges short, close to margins of coxal cavities.

Elytra slightly wider than long, length 0.70 mm, width 0.77 mm; moderately constricted and truncate at bases; each elytron with two large, asetose basal foveae; discal striae long, curved, extending from outer basal foveae posteriorly to 4/5 of elytral length; humeri moderately raised, small subhumeral foveae present, thin marginal striae extending posteriorly from foveae to posterior margins of elytra. Metathoracic wings fully developed.

Mesoventrite short, laterally fully demarcated from metaventrite by oblique ridges; median mesoventral foveae widely separated, originating from shared setose, transverse opening, lateral mesoventral foveae large and setose, broadly forked internally; prepectus massive, collar-shaped; mesoventral intercoxal process short, apically blunt; marginal striae complete. Metaventrite broadly and distinctly impressed at middle and densely setose at lateral portions of impression, with large, setose lateral mesocoxal foveae and pair of smaller, setose lateral metaventral foveae, metaventral intercoxal process with small and narrow split at middle.

Legs moderately elongate; protibia with disc-like impression at apical 2/5 of ventral surface (Fig. 1D); mesotibia with distinct spine at apex (Fig. 1E); mesofemur widened to middle.

Abdomen slightly narrower than elytra, widest at lateral margins of tergite 1(IV), length 0.62 mm, width 0.71 mm; lacking modifications. Tergite 1 (IV) longer than 2–4 (V–VII) combined, setose basal sulcus separated by mediobasal and one pair of basolateral foveae, lacking discal carinae; tergites 2–4 (V–VII) each with one pair of basolateral foveae, tergite 4 (VII) slightly shorter than 2 and 3 combined along middle, tergite 5 (VIII) semicircular, posterior margin roundly emarginate at middle. Sternite 2 (IV) with large mediobasal and broad basolateral foveae, lacking lateral carinae; midlength of sternite 2 (IV) slightly shorter than sternites 3–5 (V–VII) combined, 3–5 each with one pair of tiny basolateral foveae, sternite 6 (VIII) transverse, posterior margin sinuate, sternite 7 (IX) (Fig. 1F) suboval, weakly sclerotized, with scattered long setae along apical margin.

Aedeagus (Fig. 1G, H) 0.35 mm in length, moderately sclerotized, dorso-ventrally strongly asymmetric; median lobe with large, extended basal capsule and roundly triangular foramen, ventral stalk dorso-ventrally broadened at base and with pointed apex; dorsal lobe extremely elongate and evenly curved ventrally; parameres reduced to single broad membranous structure.

**Female.** Unknown.

#### Comparative notes.

The male of this species exhibits morphological similarities to *A.nepaeformis* Yin, 2022 distributed in Cona and Nyingchi counties, particularly in the general appearance and position of sexual characters on the male protibiae. However, these two species can be readily distinguished by the structure of their aedeagi. Additionally, *A.nepaeformis* is characterized by markedly modified protibiae and significantly more elongated apical spines of the mesotibiae. In contrast, the new species displays only a subtle impression on the protibia, and the apical spine of the mesotibia is notably shorter. A total of 28 species of this genus are known from Xizang.

#### Distribution.

Southwest China: Xizang (Lhünzē County) (Fig. 4).

#### Etymology.

The name is a toponymy referring to the type locality of this species, Lhünzē County.

### ﻿ Tribasodites liangi sp. nov.

Taxon classificationAnimaliaColeopteraStaphylinidae

2444E812-21FD-518E-8713-1029052C2B41

https://zoobank.org/ED8AA4E3-AE72-4B95-98A3-1C02B340AF46

**Figure 2.**
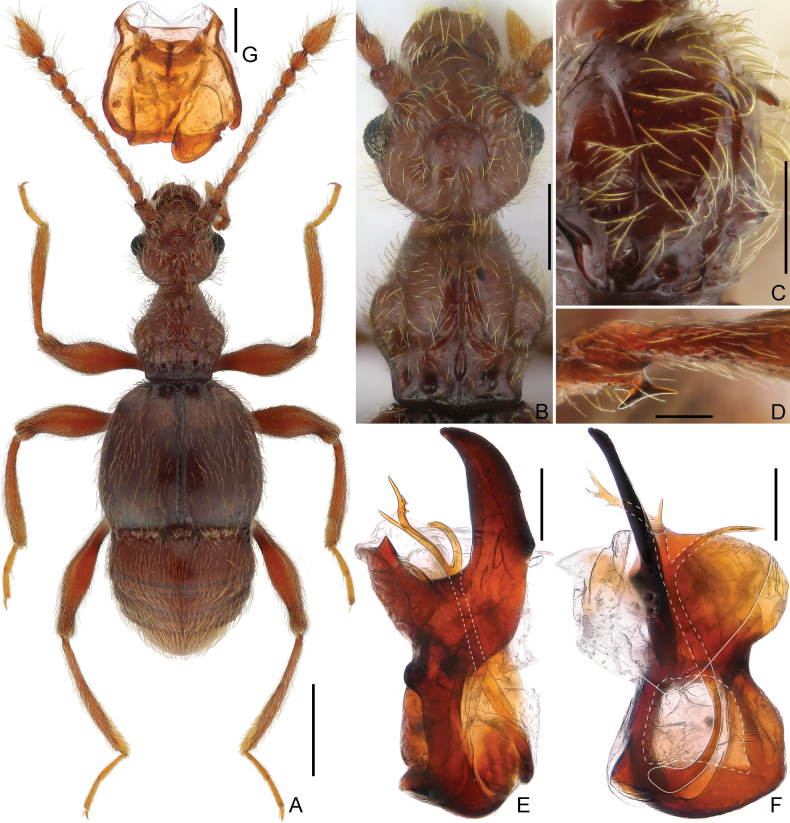
Morphology of *Tribasoditesliangi* sp. nov. (**A–F** male **G** female) **A** dorsal habitus **B** head and pronotum **C** pronotum, in dorsolateral view **D** metatrochanter **E, F** aedeagus, lateral (**E**), and dorsal (**F**) **G** genitalia. Scale bars: 0.5 mm (**A**); 0.3 mm (**B**); 0.2 mm (**C**); 0.1 mm (**D, E, F, G**).

Figs 2 , 4 Chinese common name: 梁氏脊胸蚁甲

#### Type material

(4 exx.). ***Holotype***: China: • ♂: ‘China: Xizang, Shannan, Lhünzē County, Yümai Town, Jianzhejinzhe, 28°30'21"N, 93°07'07"E, 2930 m, in wood, 5.ix.2023, Hong-Bin Liang leg., 西藏隆子县玉麦乡件哲金哲’ (SNUC). ***Paratypes***: China: • 3 ♀♀, same collecting data as for holotype (SNUC, IZCAS).

#### Diagnosis.

**Male.** Body length approximately 2.4 mm. Head subglobose, slightly narrower than pronotum; vertex with complete reversed U-shaped sulcus connecting small, asetose foveae, with distinct mediobasal carina extending from head base anteriorly to level of middle length of eyes. Antenna elongate, lacking modifications. Pronotum lacking marginal spines, with distinct median and lateral longitudinal sulci, with pairs of big discal and antebasal spines. Discal striae of elytra shallow, extending posteriorly approximately to half of elytral length. Metatrochanter with large projection on ventral margin. Aedeagus strongly asymmetric; median lobe with large basal capsule and broad foramen, ventral stalk greatly broadened at base, dorsal lobe slender and widely forked in apical portion, parameres reduced and forming single membranous structure. **Female.** Body length approximately 2.6 mm; legs simple, genitalia as in Fig. 2G.

#### Description.

**Male.** Body (Fig. 2A) 2.42 mm long, reddish-brown, elytra slighter darker, with tarsi and mouthparts lighter in color. Dorsal surface of body covered with relatively long pubescence.

Head (Fig. 2B) subglobose, rounded at base, slightly wider than long, length 0.49 mm, width across eyes 0.51 mm; vertex finely punctate, with small, asetose vertexal foveae (dorsal tentorial pits), with complete, reversed U-shaped sulcus connecting foveae, mediobasal carina distinct, extending from head base anteriorly to level of eye midlength, lateral carinae complete; tempora rounded; frons anteriorly fused with clypeus at middle, anterolaterally with thin oblique carinae; area between moderately raised antennal tubercles weakly impressed; clypeus with smooth surface, entire anterior margin strongly carinate and moderately raised; ocular-mandibular carinae complete; Venter with small gular foveae (posterior tentorial pits) originating from shared transverse opening, with thin median carina extending from opening anteriorly to mouthparts. Compound eyes moderately prominent, composed of approximately 43 small ommatidia. Antenna elongate, length 1.30 mm, indistinct club loosely formed by slightly enlarged apical three antennomeres; antennomere 1 thick, subcylindrical, antennomeres 2–8 each slightly elongate, 8 shortest, 9 wider and longer than 8, 10 wider and slightly longer than 9, 11 largest, slightly longer than 9 and 10 combined (25:22), subconical, anterolateral margin slightly impressed.

Pronotum (Fig. 2B) slightly longer than wide, length 0.48 mm, width 0.44 mm, widest at middle; lateral margins lacking spines, rounded, convergent basally and parallel at basal 1/5; disc convex, finely punctate, distinct median longitudinal sulcus with slightly carinate margins, posteriorly confluent with oval antebasal impression and short mediobasal carina, with pair of thin lateral longitudinal sulci, and pairs of discal and antebasal spines (Fig. 2C); lateral antebasal foveae distinct and setose; with distinct outer and inner pair of basolateral foveae. Prosternum with basisternal (precoxal) portion longer than procoxal rests, with large lateral procoxal foveae; hypomeral grooves obliquely extending from base anteriorly to half-length of hypomera, with lateral antebasal hypomeral impression, hypomeral ridges close to margins of coxal cavities, extending anteriorly to meet hypomeral grooves.

Elytra slightly wider than long, length 0.75 mm, width 0.90 mm; each elytron with three moderately large, asetose basal foveae; discal striae extending posteriorly from outer basal foveae to half of elytral length; humeri moderately prominent, subhumeral foveae present, carinate marginal stria extending from foveae to posterior margins of elytra.

Mesoventrite short, demarcated from metaventrite by oblique ridges; median mesoventral foveae broadly separated, originating from shared setose, transverse opening, large lateral mesoventral foveae forked internally; prepectus massive, collar-shaped; mesoventral intercoxal process short, apically acute; marginal striae complete. Metaventrite prominent admesally, inclined towards middle, with well-developed lateral mesocoxal and two lateral metaventral foveae, metaventral intercoxal process with small and narrow split at middle.

Legs elongate; procoxa with exceptionally long seta at base; mesotrochanter with tiny ventral tubercle; metatrochanter (Fig. 2D) with distinct ventral projection greatly broadened at apex.

Abdomen widest at lateral margins of tergite 1 (IV), length 0.76 mm, width 0.80 mm. Tergite 1 (IV) more than twice as long as 2 (V), setose basal sulcus separated by two mediobasal and two pairs of basolateral foveae, with pair of short discal carinae, inner marginal carinae thin and complete, outer carinae present for basal 1/2; tergite 2 (V) slightly longer than 3 (VI), 4 (VII) as long as tergites 2 and 3 combined; tergites 2–4 (V–VII) each with one pair of small basolateral foveae, tergite 5 (VIII) semicircular, transverse, posterior margin roundly emarginate at middle. Sternite 2 (IV) with one pair of small mediobasal and three pairs of basolateral foveae, lacking lateral carina; midlength of sternites 2–4 (IV–VI) gradually shorter, 5 (VII) slightly longer than 4, 3–5 lacking basolateral foveae, sternite 6 (VIII) transverse, posterior margin broadly emarginate at middle.

Aedeagus (Fig. 2E, F) elongate, length 0.54 mm, dorso-ventrally strongly asymmetric; median lobe with large basal capsule and broad foramen, ventral stalk broadest at base, narrowing towards apex; dorsal lobe long and broadly forked apically, left fork (orientation according to Fig. 2F) split into four spines at apex, and one longer spine before middle; parameres fused, broad and flattened, membranous.

**Female.** Similar to male in external morphology; antenna slightly shorter, simple, legs lacking tubercles or projections; each compound eye composed of approximately 38 ommatidia; humeri weakly raised; metathoracic wings fully developed. Measurements (as for male): body length 2.56–2.60 mm; length/width of head 0.50–0.51/0.52–0.53 mm, pronotum 0.52–0.53/0.51–0.52 mm, elytra 0.72–0.80/0.91–0.92 mm; abdomen 0.83–0.90/0.81–0.83 mm; length of antenna 1.24–1.27 mm; genitalia (Fig. 2G) moderately sclerotized, broad, maximum width 0.32 mm.

#### Comparative notes.

This species closely resembles several congeners from Xizang due to the presence of discal and antebasal spines of the pronotum, as well as the simple antennae of the male. However, *Tribasoditesliangi* sp. nov. is distinguished by the lack of marginal spines of the pronotum and its uniquely structured aedeagus, characterized by an elongate, slender, and apically serrate dorsal lobe.

#### Distribution.

Southwest China: Xizang (Lhünzē County) (Fig. 4).

#### Etymology.

This species is named after Hong-Bin Liang, collector of the type series.

### ﻿ Tribasodites yumaicus sp. nov.

Taxon classificationAnimaliaColeopteraStaphylinidae

BDCDF682-1C95-5883-B762-7CA486543DC5

https://zoobank.org/A01F5C1C-D138-4F31-B23C-2318757CEA2C

**Figure 3.**
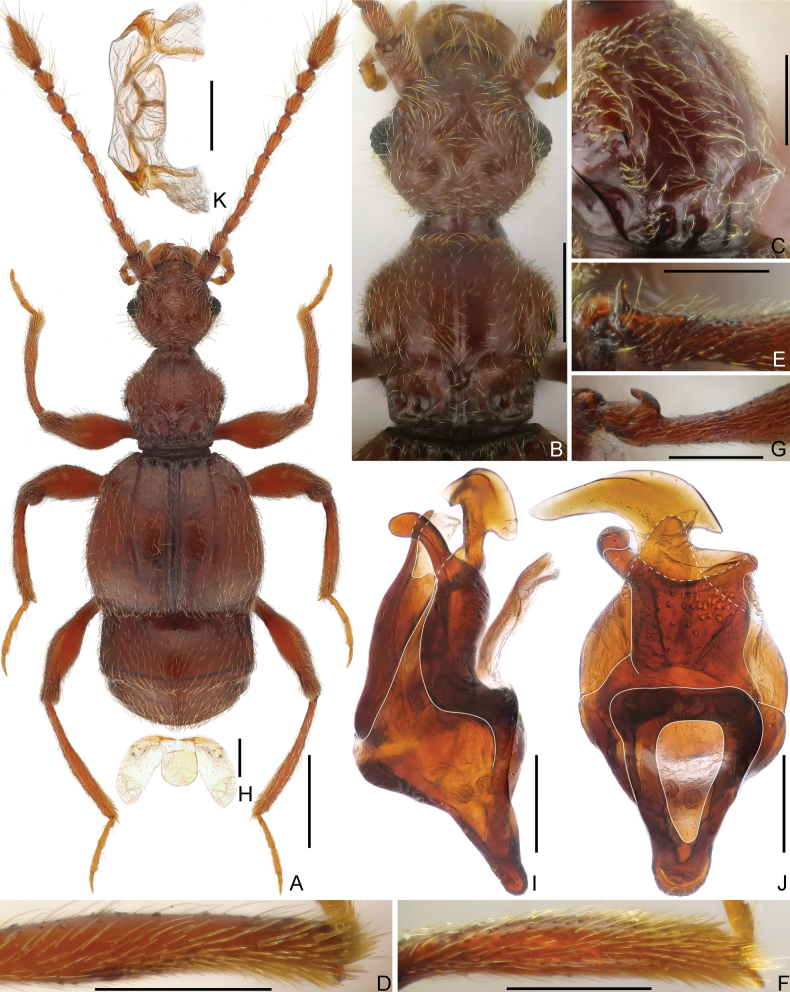
Morphology of *Tribasoditesyumaicus* sp. nov., (**A–J** male **K** female) **A** dorsal habitus **B** head and pronotum **C** pronotum, in dorsolateral view **D** protibia **E** mesotrochanter **F** mesotibia **G** metatrochanter **H** sternite 7 (IX) **I, J** aedeagus, lateral (**I**), and ventral (**J**) **K** genitalia. Scale bars: 0.5 mm (**A**); 0.3 mm (**B**); 0.2 mm (**C, D, E, F, G**); 0.1 mm (**H, I, J, K**).

**Figure 4.**
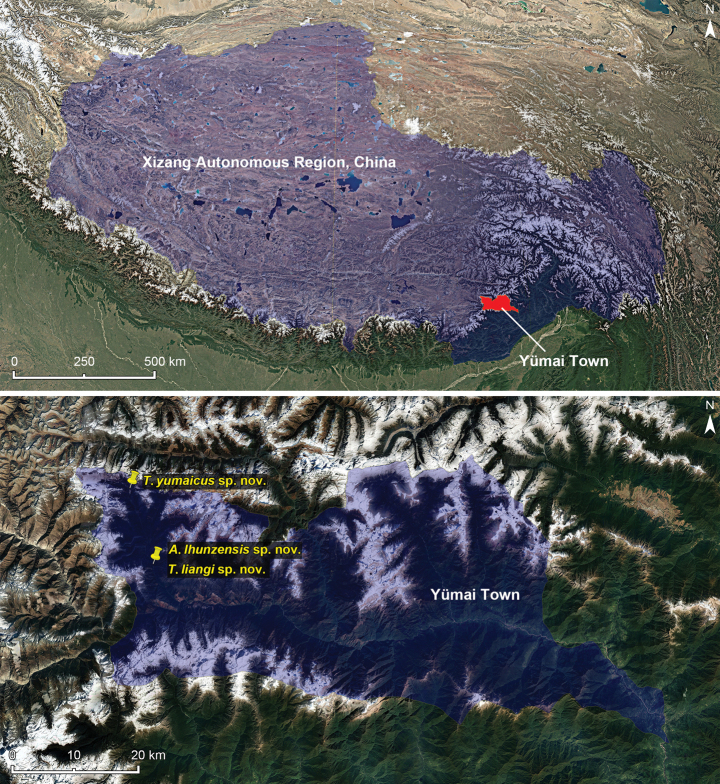
Distribution of the three species in Yümai Town, Xizang. **A** location of Yümai Town in Xizang **B** distribution of *Arthromelodeslhunzensis* sp. nov., *Tribasoditesliangi* sp. nov. and *Tribasoditesyumaicus* sp. nov. in a north-south valley located at western Yümai Town.

Figs 3 , 4 Chinese common name: 玉麦脊胸蚁甲

#### Type material

(6 exx.). ***Holotype***: China: • ♂: ‘China: Xizang, Shannan, Lhünzē County, pass to Yümai Town, 28°38'18"N, 93°4'23"E, 3660 m, under stone, 4.ix.2023, Hong-Bin Liang leg., 西藏隆子县玉麦北1公里处观景台’ (SNUC). ***Paratypes***: China: • 1 ♂, 4 ♀♀, same collecting data as for holotype (SNUC, IZCAS).

#### Diagnosis.

**Male.** Body length approximately 2.5 mm. Head roundly rectangular, slightly narrower than pronotum; vertex with shallow reversed U-shaped sulcus connecting small, asetose foveae, with distinct mediobasal carina extending from head base anteriorly to slightly below level of middle length of eyes. Antenna elongate, lacking modifications. Pronotum with small, acute marginal spines, with two pairs of antebasal spines. Discal striae of elytra long, extending posteriorly to apical 4/5 of elytral length. Protibia with small apical tubercle; mesotrochanter with distinct ventral spine, mesotibia with short spine at apex; metatrochanter with hook-like projection. Aedeagus strongly asymmetric; median lobe with subtriangular basal capsule and foramen, ventral stalk split to two projections, dorsal lobe plate-like. **Female.** Body length approximately 2.2–2.4 mm; legs lacking spines, tubercles, or projections, genitalia as in Fig. 3K, greatly transverse.

#### Description.

**Male.** Body (Fig. 3A) length 2.51–2.52 mm; color reddish-brown, tarsi and mouthparts lighter. Dorsal surface of body covered with relatively dense pubescence.

Head (Fig. 3B) roundly rectangular, truncate at base, slightly wider than long, length 0.47–0.50 mm, width across eyes 0.50–0.51 mm; vertex finely punctate, with small, asetose vertexal foveae (dorsal tentorial pits), with shallow, complete, reversed U-shaped impression connecting foveae, mediobasal carina distinct, extending from head base anteriorly to below level of eye midlength, lateral carinae present only posterior to antennal tubercles; tempora rounded; frons anteriorly fused with clypeus at middle, anterolaterally with thin oblique carinae; area between moderately raised antennal tubercles weakly impressed; clypeus with smooth surface, entire anterior margin strongly carinate and moderately raised; ocular-mandibular carinae complete. Venter with small gular foveae (posterior tentorial pits) originating from shared oval opening, with weak median carina present only for short distance near mouthpart. Compound eyes moderately prominent, composed of approximately 45 small ommatidia. Antenna elongate, length 1.54–1.60 mm, indistinct club formed by slightly enlarged apical three antennomeres; antennomere 1 thick, subcylindrical, 2–7 each slightly elongate, 3 and 8 shortest, 9 slightly wider and longer than 8, 10 wider than 9, subconical, 11 largest, longer than 9 and 10 combined (34: 27), subfusiform, anterolateral margin impressed.

Pronotum (Fig. 3B) slightly longer than wide, length 0.56–0.57 mm, width 0.53–0.54 mm, widest slightly anterior to middle; lateral margins rounded, convergent basally and parallel at basal 1/5, with pair of small, acute spines; disc slightly convex, finely punctate, distinct median longitudinal sulcus with slightly carinate margins, posteriorly confluent with oval antebasal impression and distinct mediobasal carina, with pair of thin lateral longitudinal sulci and two pairs of antebasal spines (Fig. 3C); lateral antebasal foveae distinct and setose; with distinct outer and inner pair of basolateral foveae. Prosternum with basisternal (precoxal) portion longer than procoxal rests, with small lateral procoxal foveae; hypomeral grooves obliquely extending from base anteriorly to half-length of hypomera, with lateral antebasal hypomeral impressions, hypomeral ridges close to margins of coxal cavities, extending anteriorly to meet hypomeral grooves.

Elytra much wider than long, length 0.81–0.84 mm, width 0.95–0.97 mm; each elytron with three large, asetose basal foveae; long discal striae extending posteriorly from outer basal foveae posteriorly for 4/5 elytral length; humeri moderately prominent, subhumeral foveae present, carinate marginal striae extending from foveae to posterior margins of elytra. Metathoracic wings fully developed.

Mesoventrite short, demarcated from metaventrite by oblique ridges; median mesoventral foveae moderately separated, originating from shared setose, transverse opening, large lateral mesoventral foveae unforked internally; prepectus massive, collar-shaped; mesoventral intercoxal process short, apically acute, marginal striae complete. Metaventrite prominent admesally, inclined towards middle, with well-developed lateral mesocoxal and two lateral metaventral foveae, metaventral intercoxal process with small and narrow split at middle.

Legs elongate; procoxa with exceptionally long seta at base, protibia (Fig. 3D) with small apical tubercle; mesotrochanter (Fig. 3E) with distinct ventral spine, mesotibia (Fig. 3F) with small spine at apex; metatrochanter (Fig. 3G) with hook-like projection.

Abdomen widest at lateral margins of tergite 1 (IV), length 0.76–0.78 mm, width 0.81–0.84 mm. Tergite 1 (IV) more than twice as long as 2 (V), thin basal sulcus interrupted by one pair of mediobasal and one pair of basolateral foveae, with pair of short discal carinae, oblique inner marginal carinae thin and complete, outer carinae present for basal 1/2; tergite 2 (V) slightly longer than 3 (VI), 4 (VII) shorter than tergites 2 and 3 combined, 2–4 (V–VII) each with one pair of small basolateral foveae; tergite 5 (VIII) semicircular, transverse, posterior margin roundly emarginate at middle. Sternite 2 (IV) with one pair of mediobasal and three pairs of basolateral foveae, lacking lateral carina; midlength of sternites 2–4 (IV–VI) gradually shorter, 5 (VII) slightly longer than 4, 3–5 each with two pairs of small basolateral foveae, sternite 6 (VIII) transverse, posterior margin broadly emarginate at middle, sternite 7 (IX) (Fig. 3H) membranous, composed of pair of lateral lobes and one oval median plate.

Aedeagus (Fig. 3I, J) length 0.45 mm, dorso-ventrally strongly asymmetric; median lobe with subtriangular basal capsule and elongate foramen, ventral stalk divided into two parts in apical portion, one lobe short, rounded at apex, another greatly expanded at apex, dorsal lobe broad, plate-like, surrounding ventral stalk, parameres fused and reduced to ventral membrane.

**Female.** Similar to male in external morphology; antenna slightly shorter, simple, legs lacking tubercles, spines or projections; each compound eye composed of approximately 35 ommatidia; humeri weakly raised; metathoracic wings fully developed. Measurements (as for male): body length 2.21–2.38 mm; length/width of head 0.46–0.51/0.47–0.52 mm, pronotum 0.51–0.56/0.48–0.55 mm, elytra 0.76–0.84/0.87–0.96 mm; abdomen 0.56–0.61/0.67–0.71 mm; length of antenna 1.37–1.56 mm; genitalia (Fig. 3K) slightly sclerotized, greatly transverse, maximum width 0.28 mm.

#### Comparative notes.

This species closely resembles several congeners from Xizang due to the similarly structured aedeagus, i.e., dorsal lobe of the aedeagus broadened and encircling the ventral stalk. However, *Tribasoditesyumaicus* sp. nov. can be readily distinguished by the simple male antennae and the unique configuration of the aedeagus. With the addition of the two new species described here, the total number of known species of this genus occurring in Xizang has increased to 15.

#### Distribution.

Southwest China: Xizang (Lhünzē County) (Fig. 4).

#### Etymology.

The name is a toponymy referring to the type locality of this species, Yümai Town.

## Supplementary Material

XML Treatment for Arthromelodes lhunzensis

XML Treatment for Tribasodites liangi

XML Treatment for Tribasodites yumaicus

## ﻿Additional information

### Author ORCIDs

Zi-Wei Yin https://orcid.org/0000-0001-6659-9448

### Data availability

All of the data that support the findings of this study are available in the main text.
